# Facilitators and Barriers to the Implementation of a School-Based Intervention for Anxiety

**DOI:** 10.1007/s10578-023-01615-9

**Published:** 2023-11-16

**Authors:** Gemma K. Brown, Jane Owens, Cathy Richards, Simona Di Folco, Matthias Schwannauer

**Affiliations:** 1 Child and Adolescent Mental Health Service, Royal Edinburgh Hospital, NHS Lothian, Edinburgh, UK; 2 Section of Clinical and Health Psychology, School of Health in Social Science, The University of Edinburgh, Doorway 6, Teviot Place, Edinburgh, EH8 9AG UK; 3 Child and Adolescent Mental Health Service, NHS Lanarkshire, 14 Beckford Street, Hamilton, ML3 OTA UK

**Keywords:** School-based, Implementation, Process evaluation, Cognitive behavioural, Anxiety

## Abstract

School-based cognitive behavioural interventions for anxiety are found to be effective, but there is a lack of research on their implementation in real world settings. The current study aims to explore the facilitators and barriers to the implementation of a school-based intervention for anxiety through a qualitative process evaluation. Evaluation of the implementation of Let’s Introduce Anxiety Management (LIAM), a six-session school-based cognitive behavioural intervention, was conducted. LIAM was implemented by non-mental health professionals trained and coached on the model. Semi-structured interviews with stakeholders (N = 15) were analysed with grounded theory and framework analysis. Forty-one practitioners were trained and coached on LIAM, with thirty-five children and young people receiving the intervention. Facilitators (e.g. systemic collaboration, self-efficacy and an enabling context) and barriers (e.g. the exclusivity of the intervention and a lack of systemic understanding) to implementation emerged as themes. Implementing school-based interventions is complex and requires the involvement of multiple stakeholders.

## Introduction

Early intervention for childhood anxiety has been identified as a key priority of the Scottish Government’s Mental Health Strategy 2017–2027 [1]. This national policy sets out the need to upskill the workforce in universal settings and increase the availability of evidence based, low-intensity interventions outside ofspecialist mental health services.

A meta-analysis of one hundred and eighteen studies examined manualised school-based programmes for the prevention of anxiety and depression in children and young people (CYP) and found a small, positive effect post intervention [2]. However, translating such research findings to real-world settings is acknowledged to be a complex process [3] and overall, the successful implementation of evidence-based interventions in school settings is thought to be low [3]. A recognition of this ‘science to service gap’ [4] has led to a rapid growth in the field of implementation science [5, 6] and process evaluation. While multiple models of these processes exist [7–10], implementation science is broadly concerned with “how an intervention is put into practice, how it operates to achieve its intended outcomes, and the factors that influence these processes” (11, p.9).

Implementation Science suggests that training in a specific intervention is necessary but not solely sufficient for successful delivery [4]. In order to sustain the successful delivery of an intervention factors such as access to on-going expertise and support, resources and a supportive organisational context are required.

Literature on the implementation of interventions within the school setting has reported on the characteristics of the intervention, client, individual implementer and wider system, alongside the importance of preplanning and on-going support in line with implementation frameworks [4, 11, 12]. A review of health promotion programmes in UK schools acknowledged the complexity of this process and noting that these factors do not occur in isolation [13]. Intervention specific factors include training and performance feedback as well as the acceptability of the intervention. Individual factors include self-efficacy, professional burnout and professional support alongside skill, attitude, engagement and beliefs but may be separated into professional characteristics, perceptions and attitudes regarding the intervention and their psychological characteristics [11, 12, 14]. Organisational factors include the attitudes, beliefs and behaviours of managers, administrators and other stakeholders as well as resource, policy and procedures [12, 15, 16]. A systematic review of implementing brief interventions, although not specifically school or Cognitive Behavioural Therapy (CBT) based, echoed the above implementation factors and particularly highlighted the importance of ongoing support in the form of skills coaching sessions [17]. There is limited literature on how these factors occur in relation to CBT school-based interventions but previous studies have supported the structure of implementation frameworks [18, 19]. Greater organisational structure, peer support and administrative support allowed sites to overcome barriers to implementation of a trauma-focused intervention [18], whilst the complexity of the difficulties being treated were noted as a barrier to implementation of an intervention for anxiety [19].

Despite the growing demand for evidence based mental health interventions in schools, there is a limited focus on how to successfully implement these outside of a research context. Previous studies on school-based interventions for mental health and well-being are primarily restricted to effectiveness and efficacy research trials and do not report on implementation [20] despite this being noted as impacting outcome [15, 21]. Existing literature on school-based implementation processes has focused on a broad range of health promotion programs and there is a paucity in the literature on the processes involved in bringing mental health interventions to a wider audience [11]. In addition, existing literature often focuses on implementation factors already identified in the literature [22] with possible ‘hidden mechanisms’ going undetected and therefore leading to potential bias [13].

The aim of the current study is to explore the implementation of an evidence-based intervention for anxiety (Let’s Introduce Anxiety Management (LIAM)) in school settings. The first stages of implementation, described by Fixsen et al. [7] as the ‘installation’ and ‘initial implementation’ phases of implementation, will be focused upon using qualitative data. Interviews with stakeholders will explore barriers and facilitators to the implementation with data relating to the reach of the project providing a context in which to consider these factors. It is hoped that, through this, the current study will inform and improve the implementation of future school-based mental health interventions. The current study reports on the multi-agency installation and initial implementation of the intervention during the school year 2017 to 2018.

## Method

### Study Design

The process evaluation utilises qualitative data consisting of individual interviews. Data on the reach of the intervention was collected to provide context on implementation outcomes. As prior constructs and knowledge were imposed on the data through the predefined model of LIAM training and coaching, a Social Constructivist version of Grounded Theory [23] was used in conjunction with framework analysis [24]. This allowed for both a priori issues and emergent themes grounded in data to simultaneously guide analysis within the systems in which they occur [10].

### Participants

All project stakeholders, including service managers and LIAM practitioners, were eligible and invited to take part in individual interviews. A total of 15 participants took part in the study consisting of School Nurses (n = 7), education staff (n = 5) and managers (n = 3). Education staff included Pupil Support Officers (PSO; n = 4), and an additional support for learning teacher (n = 1), while managers included an Educational Psychologist (n = 1), A School Nurse Manager (n = 1) and Clinical Psychologist (n = 1). Demographics for participants are summarised in Table 1.

**Table 1:** Participant characteristics

Participant	Profession	Months post initial training	Practitioner delivery of LIAM on-going at interview	Interview length (mins)	Reference
1	School Nurse	0	No	55	SN1
2	School Nurse	1	No	34	SN2
3	School Nurse	2	No	33	SN3
4	School Nurse	2	No	63	SN4
5	School Nurse	4	No	52	SN5
6	Manager	4	N/A	51	M6
7	Manager	5	N/A	63	M7
8	Education Staff	5	No	50	E8
9	Manager	6	N/A	47	M9
10	Education Staff	6	Yes	47	E10
11	Education Staff	6	Yes	44	E11
12	Education Staff	6	Yes	80	E12
13	School Nurse	7	Yes	38	SN13
14	School Nurse	7	Yes	36	SN14
15	Education Staff	7	Yes	69	SN15

Sampling was purposive and directed to capture a range of experiences relating to the stage of implementation, level of LIAM delivery and professional role [23]. Of those approached, two PSOs declined to take part. Theoretical sampling [25] was used to guide data collection and refine the emerging categories from initial coding and analysis [26, 27].

### Intervention

LIAM was developed by NHS Education Scotland [28] as part of an initiative to increase access to evidence based early intervention approaches outside specialist mental health settings. The aims of the project are displayed in Table 2.

**Table 2:** Aims of let’s introduce anxiety management

Short Term	Long Term
Systemic: Promote psychological awareness in this area and enable workers in children’s services to recognise and respond to anxiety	Systemic: Develop pathways that increase access to psychologically informed care and interventions for the large groups of CYP could benefit from this
Practitioner: Improve skills of those professionals who might have contact with anxious children. Practitioners develop manualised evidence-based CBT informed techniques and an understanding of anxiety	Practitioner: Upskill the broader workforce, outside of tier 3 CAMHS, in children’s services across Scotland. Develop self-sustaining systems of training, supervision, coaching and implementation to include outcome monitoring
CYP: CYP are identified and receive evidence-based treatment with fidelity CYP learn strategies to manage anxiety	CYP: Better outcomes, early intervention, reduce impact of mental health difficulties

LIAM is a manualised, Cognitive Behavioural Therapy (CBT) informed intervention for low level childhood anxiety. The intervention consists of six, individually delivered, modules (Table 3).

**Table 3:** Overview of the intervention, Let’s Introduce Anxiety Management

Module	Content
1.Psycho-education	Normalisation, fight or flight, avoidance trap
2.Self-monitoring	Link between thoughts feelings behaviours, Feelings diaries and thermometer
3.Emotional awareness and management	Physiological response to anxiety, relaxation, distraction
4.Coping thoughts	Unhelpful thoughts, thinking styles and helpful thoughts
5. Exposure	Graded exposure through fear ladder and thermometer
6.Reinforcement	Rewards, record of achievement, maintaining progress

Adapted from the session guide in LIAM Trainer’s Manual (NES, 58)

LIAM practitioners receive 2 days of face-to-face training and complete an additional e-learning package before delivering LIAM to CYP. Once trained, LIAM practitioners are required to attend fortnightly group coaching sessions in order to triage referrals, support skill development, monitor risk and enhance model fidelity. Training and coaching are provided by Clinical Psychologists based in local NHS Child and Adolescent Mental Health Services (CAMHS).

Initial implementation planning identified 2 key professional groups for LIAM delivery. School Nursing, whose developing role included an enhanced focus on mental health and well-being [29] and Pupil Support Officers (PSOs); a recently developed school-based role focusing on supporting the emotional well-being of CYP.

### Procedure

Potential participants were made aware of the current study during LIAM training or the subsequent coaching sessions via the researcher (GB) or LIAM coach (JO). Those who expressed an interest in participating in the study were contacted to arrange a time to meet with the researcher (GB). A participant information sheet was provided and written consent was obtained prior to the interview. Ethical approval was obtained from a local authority and The University of Edinburgh, School of Health in Social Science.

### Measures

#### Reach of LIAM

In order to provide context to the facilitators and barriers of implementation [10], data relating to the reach of the project was collected. This included the number of practitioners trained, attending coaching and delivering LIAM as well as the number of CYP receiving the intervention.

### Interviews

Interviews were conducted over an eight-month period following LIAM training. The interviews followed an open, in-depth format and flexible administration in response to the participant’s concerns. The aim of the interviews was to understand the process of implementation and identify facilitators and barriers to doing so. Initial questions were around the participant’s role, their perception of CYP’s needs and how LIAM would work alongside these. This was used to create a discussion led by the participants concerns rather than specific questions around barriers and facilitators to implementation. The interview format evolved throughout sampling as themes emerged through initial coding, the use of memos and reflective discussion within supervision. Probes were used when appropriate, and participants were encouraged to share autobiographical memories in order to gain rich data [23]. Interviews ranged from 33 to 80 min (M = 50.8; SD = 13.52) and were audio recorded then transcribed verbatim from a digital file. Data was anonymised at the point of transcription and stored and analysed using NVivo 11.

#### Quality

The current study considered the following core principles presented in the framework by Yardley [30, 31]: sensitivity and context; commitment and rigor; coherence and transparency and impact and importance. Memos, including the first author’s notes and reflections, were used throughout the research process to ensure transparency and sensitivity to the context of the research process. Memos documented emerging themes and highlighted potential biases from the researcher [23] while discussion and review of the coding within supervision ensured interpretation was not confined to a single perspective. An audit trail of the research process was kept linking the data to final analysis.

The researcher’s role as an active agent in the collection and interpretation of the data was considered [23] in analysis. The lead researcher was aware of their own preconceptions, such as knowledge of existing implementation frameworks and CBT based interventions for CYP. In addition, the researcher had involvement in the implementation of LIAM outside the research process (e.g. delivering training, attending coaching sessions or stakeholder events) and experience delivering low-intensity CBT based interventions with CYP. Participants were also aware of the lead researcher’s connection to the LIAM coach and co-author (JO as placement and research supervisor for GB), introducing potential for responses to be biased by social desirability. The impact of the researcher on participants and interpersonal dynamics were considered in analysis through reflection in supervision and use of memos.

### Analysis

Analysis of the interviews followed the grounded theory approach [23]. Memos were included in the analytic process to ensure transparency in interpretation. Line-by-line coding of the raw data, reflecting the language of participants, was completed to identify key descriptive concepts grounded in the data and reduce the imposition of pre-analytic assumptions on analysis. In a reductive process, low level categories emerged from initial coding and were used to generate new interview questions and a conceptual understanding of the data.

Subsequent interviews employed theoretical sampling to refine emerging high-level categories. This process was repeated until theoretical sufficiency occurred. Theoretical sufficiency [31] was sought rather than theoretical saturation [27] to account for the on-going nature of the implementation of the intervention and possibility of changes in perspective during analysis.

Constant comparison across interviews was used to examine the relationships between categories and facilitate the generation of theory alongside the exploration of negative cases to add depth to analysis and examine diversity and contrasts in the data. Abstraction from the data and theoretical categories were repeatedly examined until the data was represented in the most fitting way. Diagrams and memo writing accompanied clustering of data into the framework [23]. A framework of facilitators and barriers across different stakeholders was used as a tool to explore themes within the context of the intervention and the systems in which it was implemented.

## Results

### Sample Characteristics

#### Reach of the Intervention

Between October 2017 and January 2018, LIAM training was attended by 41 practitioners (58.5% School Nurses, 34.1% Pupil Support Officers, 7.3% Other Education Staff) By June 2018, 33 (80.4%) of these continued to attend coaching and 19 practitioners (46.3%) were delivering LIAM to CYP.

In total, 53 CYP were identified and consented to LIAM during the period of the current study. Of these, 35 (66.0%) CYP started the LIAM programme with plans for the remaining 16 (30.2%) to begin LIAM following the school summer holiday. Of the 25 CYP who concluded the intervention prior to June 2018, 88% (n = 22) completed the intervention in full and 12% (n = 3) did not complete due to not engaging.

#### Qualitative Data: Interviews

**Fig. 1:**
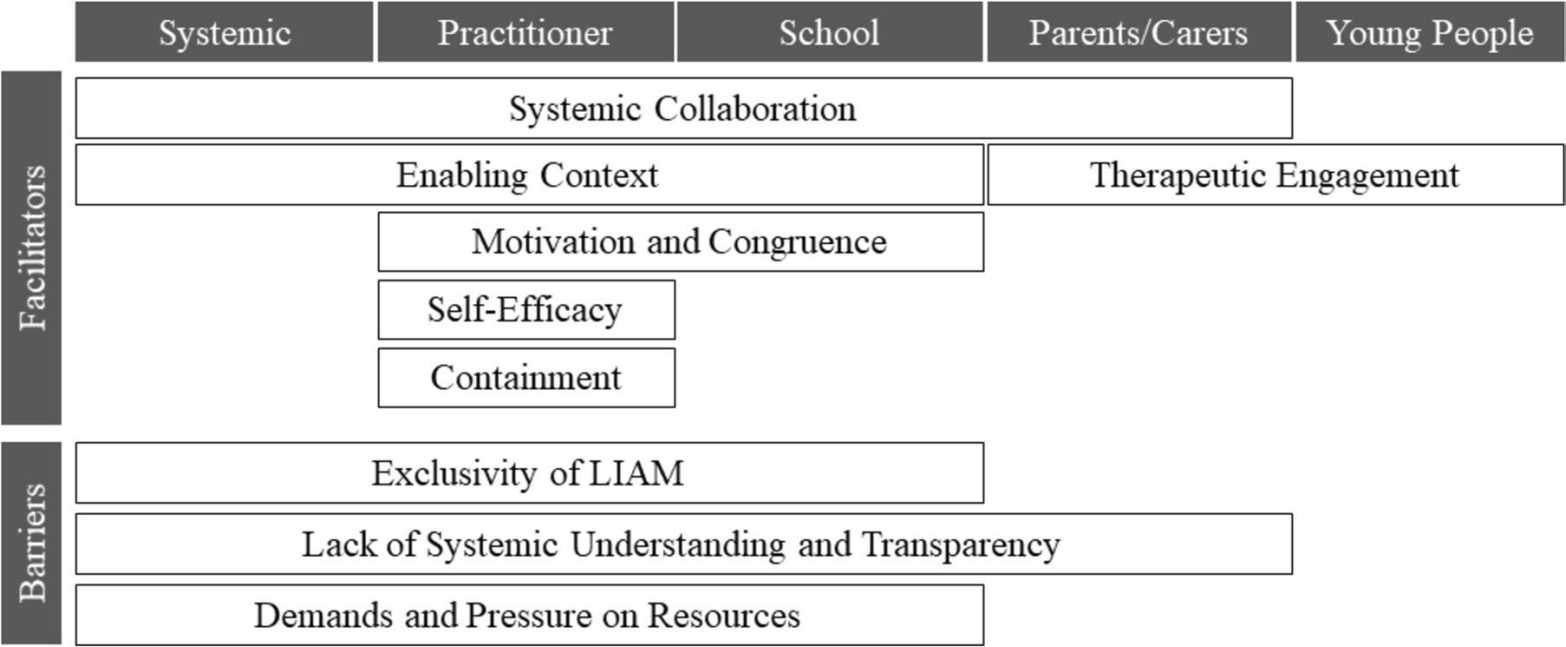
Facilitators and Barriers to the implementation of LIAM

Figure 1 displays the key categories which arose from exploration of the facilitators and barriers to the implementation of LIAM, a school based cognitive behavioural informed intervention for CYP.

Themes are presented in the framework of facilitators and barriers alongside the different stakeholder levels (systemic, practitioner, school, parents and CYP). Table 4 displays a more detailed diagrammatic representation of emerging sub-themes. Themes are illustrated below with brief quotes referencing the participant in brackets (e.g. SN5 is school nurse, participant 5).

**Table 4:** Themes and sub-themes as facilitators and barriers to implementation of LIAM

Facilitator	Barrier
*Systemic collaboration* Multi-agency pathways National and local policy Established and/or positive relationship with schools Whole school approach Practical support from schools Parental involvement Sharing progress and recommendations	*Exclusivity of LIAM* Restrictive criteria Limited scope of LIAM Personally managing rejected referrals
*Enabling context* Early intervention is a priority Fit with professional changes Pre-implementation preparation Investment in LIAM Tolerance of Pilot Parental support for LIAM	*Demands and pressure on resources* Limited resources in public sector Difficulties accessing other services LIAM is time intensive Unexpected and competing priorities Variation in job role
*Motivation and congruence* Making a difference for CYP LIAM fits with wider role Belief LIAM will be effective	*Lack of systemic understanding and transparency* Different perspectives from health and education Uncertainty around on-going role changes Not having a shared understanding of CYP needs Lack of understanding of project aims Negative beliefs about school from parents
*Self-efficacy* Relevant experience Selecting and using resources	
*Containment and encouragement* Coaching Peer support Managerial support Working in level of expertise	
*Therapeutic engagement* Appropriate and accessible delivery Embedding services in schools Therapeutic relationship	

### Explanation of Theoretical Categories

#### Facilitators

##### Systemic Collaboration

This emerged as a facilitative higher-level theme across stakeholders. It involved taking a multi-agency approach to pathway development at a systemic level, with participants reflecting on collaboration between different professionals and systems (i.e. education and health) in relation to service provision.

An established, positive relationship with schools, and in particular, the senior management team, emerged as key to facilitating implementation. Facilitative relationships between practitioners and schools, notably teachers and guidance staff, were promoted through visibility and their length of time working in a school and was crucial for the identification of CYP who may benefit from LIAM.

In addition, pragmatic support from schools such as obtaining resources and contacting families were seen as facilitative for SNs as a visiting service. Practitioners also discussed the importance of collaborating with teachers and parents, especially of primary school children, to share the progress of LIAM for CYP and any additional concerns.

##### Enabling Context

Presented across stakeholders; for this theme participants described experiencing a sense of openness and support for LIAM, and early intervention as a priority. LIAM was seen as having good fit with wider professional changes and priorities as well as with national and local policy.

In addition, managers talked about undertaking pre-implementation preparation during the exploration and installation phases of implementation leading to “readiness” (M6) in the system at management level as well as available resources. The investment in LIAM demonstrated a commitment to practitioners and promoted a sense of confidence and value in relation to delivery. Comparing LIAM to previous training, participants reported that a lack of support can lead to less investment from practitioners.

Tolerance towards the pilot status of LIAM also emerged from the interviews as a sub-theme within the “Enabling Context.” Participants were open to working collaboratively with LIAM stakeholders to tackle barriers to implementation and contributing to project development, as well as working with aspects of LIAM they were unsure of. Finally, parental support for intervention was also seen as an important in order to create an enabling context facilitating engagement with CYP and application of the intervention at home**.**

##### Motivation and Congruence

All participants reported feeling positive towards LIAM and supportive of the project. Schools and practitioners identified LIAM as being beneficial for both the school and CYP. Practitioners discussed that mental health and wellbeing is a large part of their current job role and, therefore, LIAM was congruent with that. Participants also acknowledged that, due to a lack of training prior to LIAM, the intervention met a training need for practitioners and motivated them towards it.

LIAM was reported to be congruent with practitioners’ priorities as they reported that making a difference to CYP was the core, rewarding part of their role and motivated them towards implementation.

##### Self-Efficacy

Participants discussed the need for confidence to start LIAM implementation and self-efficacy was also reported to be facilitative in building relationships with new teams. In particular, self-efficacy was relevant to the use of routine outcome measures and resources following a delay in the practitioner pack being provided. Having previous relevant professional experience was connected to increased self-efficacy alongside different educational backgrounds and completing LIAM cases, whereas where there was gap between training and LIAM delivery with CYP, practitioners reported concerns around losing confidence.

##### Containment and Encouragement

Participants reflected that the format and level of the training was appropriate and beneficial for their wider staff team, facilitating implementation. Although training contained a lot of information, the on-going support of coaching was a key facilitator and supported practitioners to overcome the barriers to implementation. Participants reflected that coaching containined anxiety about delivery, maintained the momentum of implementation and allowed practitioners to build confidence. The consistency of coaching sessions, protected time for them, as well as the supportive relationship with the coach, helped to consolidate information and to prevent drift. This improved consistency and ensured safe delivery of the intervention.

Within coaching, SNs discussed the management of referrals and this led to greater awareness of when LIAM was not appropriate and the importance of working within their area of expertise. LIAM training facilitated confidence working in mental health.

Peer support through coaching groups also emerged as facilitative. Informal support was provided in relation to sharing ideas, good practice, normalising experiences of delivery as well as considering a wider range of resources in the educational setting.

Although aspects of management and support were discussed by 10 participants, themes did not emerge at a management stakeholder level. “Managerial support,” including job role development, good communication, emotional support, and overall support for LIAM were identified by practitioners as encouraging them to overcome implementation barriers.

##### Therapeutic Engagement

As a key facilitator for working with parents and CYP, this theme was obtained through considering appropriate and accessible delivery of LIAM fitting with the needs of the CYP. For example, by considering the length and timetabling of the session, setting individualised goals and using specific learning materials (i.e. videos). In addition, practitioners discussed creating a safe, consistent space for CYP, as summarised by the sub-theme of therapeutic relationship. It was acknowledged that this could take time to develop and PSOs, as practitioners embedded in the school, were able to informally build relationships with CYP prior to implementing LIAM.

“Therapeutic engagement” also emerged as a theme at the parental level. One practitioner talked about how some parents, particularly those with their own difficulties, could find it challenging to engage with services. SNs reported that families tended to engage well with them as they were seen as a “non-threatening service” (SN5), whilst PSOs discussed how a parent’s own experiences of school could influence their beliefs around school for their child. The importance of working with them to overcome this barrier was through non-judgemental and supportive relationships was discussed.

#### Barriers

##### Lack of Systemic Understanding and Transparency

Emerging around the understanding of one another’s roles, systems, priorities and intervention aims, at a systemic level, this theme captures the different perspectives between health and education, particularly for practitioners working within this sector. Educational Psychology talked about health and education coming from different “underlying world views” (EP7) as health was focused on a deficit model while education was moving towards a more strength-based approach, in line with policy. PSOs and SNs who had not observed differences between these systems, reported LIAM was well placed as being embedded in schools and fit with their typical way of working. Several practitioners from an educational background suggested coaching around their other work on emotional well-being would be useful as well as a more educational perspective in LIAM coaching.

Identifying referrals emerged as a barrier to implementation and, on further exploration, this was attributed not only to the theme “Exclusivity of LIAM” but to the sub-themes around a lack of systemic understanding of the project aims and not having shared knowledge of CYPs’ needs. Those that were new to their school or a visiting service had to rely on others to identify CYP who may be appropriate while those that were more readily able to implement LIAM knew the pupils well, due to being established in their role and having an overview of the needs of the school.

A lack of systemic understanding of the project aims also emerged, as management were reported to not always be aware of LIAM or the details of the program, who it was appropriate for (difficulties in identifying CYP with mild anxiety) and what the intervention modality involved (decisions about referrals made within coaching with limited information).

Participants reported that the initial referral criteria in relation to the project aims was not clear with one participant reporting that it was “mis-sold” to management (ES12). Lack of understanding around the project’s aims led to inappropriate referrals initially and pressure on time made it difficult to promote understanding within the school.

In addition, a lack of understanding around job roles between different professional was a barrier for practitioners. SNs discussed frustration at their role not being known in schools and the on-going need to promote it as part of their wider professional changes whilst PSOs, who were working in a new role, discussed the difficulties becoming established in a newly created post. For practitioners, the larger professional changes led to a lack of understanding and uncertainty for SNs about their current job role.

At a parental stakeholder level, participants reported that a lack of understanding and transparency between parents and schools emerged as a barrier. For example, participants noted that parents not feeling heard by the school or holding negative beliefs about the school based on their own prior experiences.

### Exclusivity of the Intervention

Participants were concerned with the referral criteria being too restrictive or “specific” and that this had led to difficulties identifying referrals and initiating the implementation of LIAM, in particular with disagreement with the exclusion of CYP with Autism Spectrum Disorder.

Some practitioners discussed that the criteria were developed by mental health specialists and must be grounded in the evidence base, although the delay in identifying referrals was frustrating. Practitioners in the education system attributed the current referral criteria to the ‘piloting’ of the intervention and anticipated that this may change in the future, as it did not account for the context of CYP that they work with. Participants reported working with a range of needs across age groups, levels of deprivation, exposure to trauma, in relation to sexuality and systemic difficulties. The limited scope of LIAM to address diverse presentations emerged as a sub-theme barrier to implementation.

Schools were reported to primarily refer those that they were most worried about irrespective of whether they were appropriate specifically for the intervention, but there was limited scope for LIAM to address these needs. Rather, participants frequently discussed that LIAM was one part of their wider role and not “stand alone.” PSOs discussed that they offered other forms of support and interventions around mental health and well-being with CYP as part of their role while SNs discussed that they may continue working with someone even if they did not meet the criteria for LIAM and that referrals should not be so exclusive.

Practitioners also discussed difficulty excluding referrals personally. Not offering an intervention conflicted with the way that PSOs and SNs worked and they reported feeling “uncomfortable” or not “fulfilling my job role professionally” (SN14) if they rejected a referral for LIAM, particularly when presenting difficulties were not severe enough to meet CAMHS referral criteria. Practitioners reported a need to offer another service because if felt like “Letting people down a bit when you say no” (SN13). Overall, participants reported concerns that engagement with LIAM would be reduced across stakeholders if it did not meet systemic priorities. However, a manager reported that LIAM was not inappropriate for complex cases pe se but may be a small part of the input a CYP received within the provision of other services in schools where LIAM could be more integrated.

### Demands and Pressure on Resources

The impact of the “demands and pressure on resources” was discussed. While this was primarily related to time, pressure on other resources, such as accommodation, was noted in addition to the variation and volume of demands to build work in 6 weekly sessions due to holidays and timetabling.

Practitioners discussed being constrained in their ability to implement LIAM due to limited resources in the public sector. SNs discussed the challenges associated with the diversity of their role and working with a range of presentations. It could be difficult to focus on all aspects of their role, including LIAM, and noted it could be a “full time job” (SN13). PSOs discussed their changing role day to day and apprehension about implementing LIAM appropriately due to time pressure. Both SNs and PSOs reported that their time was taken away from LIAM by unexpected or competing priorities. For SNs these were primarily concerns around Child Protection and attending case conferences whilst PSOs reported that they would have to react to any difficulties arising in the school. Although needs were recognised for CYP receiving LIAM it could be hard to prioritise early intervention.

Difficulties accessing other services, such as CAMHS, due to the length of waiting times was discussed, along with the frustration of struggling to access specialist mental health services.

In addition, LIAM being time intensive emerged as a barrier for practitioners with the time required being higher than anticipated and, in particular, the work around gathering information about referrals, obtaining consent, preparing resources, use of outcome measures and attending coaching. Practitioners reported not initially building this time in and that the initial frequency of coaching had been “too frequent” (M09) when there were not cases to discuss but as delivery of the intervention had begun it was more justified. Part-time practitioners reported finding the proportion of their time on LIAM more difficult and expressed concerns around their caseload capacity. Those who overcame this barrier reported protecting time through use of a timetable, sharing this with the wider school and having support from management.

## Discussion

The study aimed to explore the facilitators and barriers to the installation and initial implementation of LIAM, a school-based cognitive behavioural intervention for anxiety involving multi-agency collaboration between health and education. The intervention aimed to create more capacity for targeted mental health interventions embedded in schools for CYP through upskilling the CYP workforce outside of CAMHS. Following training and coaching, practitioners met with CYP to implement LIAM.

Two practitioner groups implemented LIAM; SNs and education staff. Overall, few differences were identified between different practitioner groups, despite SNs not being embedded in schools, indicating similar issues with implementation across contexts. Both professional groups were under-going wider role changes that aligned with the aims of LIAM and responded in similar ways to coaching and training. While both groups reported the need to collaborate with the system, they agreed that this could be difficult if there was not a shared understanding between different professionals and within systems.

### Progress of Implementation

With respect to implementation, data on the reach of the intervention indicated that the majority of those that had completed LIAM training continued to attend coaching and had consented CYP to participate. However, a smaller proportion of practitioners had begun to deliver LIAM with CYP, suggesting that moving from training to delivering the intervention with CYP was difficult for some practitioners and barriers to implementation occurred.

Barriers also occurred prior to beginning LIAM with CYP as opposed to drop-out during the intervention. This fits with the themes that emerged as barriers from qualitative data around the lack of systemic understanding and transparency and the exclusivity of LIAM. These themes acted as barriers by making it more challenging for practitioners to identify CYP who met the referral criteria for the intervention.

### Barriers and Facilitators to Implementation

#### Characteristics of LIAM Model

Themes that emerged as barriers and facilitators to implementation complement aspects of existing implementation frameworks and previous literature. The importance of initial training and on-going support to develop and sustain intervention competencies has been extensively noted throughout the literature [7, 15, 22]. Within the current study, the data revealed that the model of training and coaching in LIAM was facilitative to implementation at the practitioner stakeholder level.

Coaching is a key competency driver within the active implementation framework [7] and was included in the initial design of the LIAM model. Interviews revealed that it facilitated implementation through containing and encouraging practitioners. This is in line with previous literature echoing the need for on-going support and expertise [22]. Coaching enabled practitioners to overcome their anxiety around delivering LIAM as well as some barriers relating to demands on time, managing resources and being ‘stuck’ with delivering the intervention. Previous research has indicated that the qualifications or training of the coach, the outcomes expected from coaching and logistics around accessing coaching can be problematic [8] but embedding coaching into the LIAM model from the exploration stage of implementation may have meant that barriers to coaching previously identified did not emerge within the current study. In addition, peer support emerged as being facilitative to implementation in line with previous research [18].

In line with previous findings, practitioners reported that adapting the delivery of LIAM to make it accessible to the individual, positive therapeutic relationships and embedding services in the school were important in engaging families [12, 13]. However, this warrants further exploration in later implementation stages when practitioners have more experiences of delivering LIAM to CYP to draw upon and compare as well as through the involvement of CYP and their parents/carers in research.

Participant responsiveness and adaptations to interventions are a key aspect of evaluating implementation [11] and, practitioners highlighted the need to use different modalities to engage CYP (e.g. video or worksheets) and adjust the frequency and length of sessions. Further examination of the impact of these adjustments on intervention outcomes was not feasible using the data obtained in the current study, but adaptations are likely to interact with the intended dose and fidelity of the intervention, although the processes by which this occurs are not established in the literature (10, 15).

#### Individual Factors

The self-efficacy of practitioners emerged as facilitative to implementation at the practitioner level, in line with previous studies [7]. Relevant previous experience led to greater self-efficacy which, in turn, led to confidence in accessing resources and implementing LIAM. While selecting staff with characteristics that are facilitative to implementation has been noted as key in previous studies [20], LIAM was delivered by existing staff members in specific staff groups, so this was not considered at an individual practitioner level, with staff selection being based on professional role more broadly. Coaching has been found to aid existing staff who may not have previous knowledge or skills, or the associated self-efficacy, within the current and previous studies [20].

The emergence of motivation and congruence as facilitative individual factors, where practitioners’ willingness to implement the intervention was influenced by their beliefs of acceptability and ease of delivery, is also congruent with existing literature [18, 32].

#### Contextual Factors

The need to take a whole system approach to implementation that includes external collaborators, whole schools and CYP and their families is documented in existing literature [12, 13]. In line with this, successful implementation was facilitated in the current study by collaborating systemically across agencies and through having good working relationships.

Previous research has noted that an individual’s willingness to implement is associated with perceptions of the presence of organisational resources and support [20] and, for LIAM, practitioners’ motivation to implement was enhanced by the investment from stakeholders at all levels. Pre-planning prior to implementation and support in the context of policy have emerged as key stages in implementation throughout the literature [7, 13, 15, 16, 22].

Despite systemic support for LIAM, pre-implementation planning, seeking to protect staff time and on-going role changes that facilitated the realignment of staff to LIAM delivery (a critical aspect of intervention installation; [7]); participants reported having limited time to deliver LIAM in the context of experiencing competing demands within their roles. The time involved with LIAM delivery, was greater than practitioners anticipated and the perceived cost–benefit in terms of delivery time required for an intervention can influence willingness to implement [20]. Support of senior management in schools is recognised as facilitative [12] and, within the current study, this aided the identification of CYP for LIAM as well as ensuring that practitioners had access to therapeutic spaces in schools and the resources needed for delivery. While higher-level managerial support was acknowledged, themes did not emerge at this level around implementation planning, rather their support was related to practitioner experience.

Disseminating the aims of an intervention and how they fit with a school’s need or ethos are key stages of implementation [13]. The current study found that where there was a lack of shared understanding around the aims and scope of LIAM throughout the system, it became difficult to identify CYP for whom LIAM may have been appropriate.

In addition, to afford ‘buy in’ at an organisational level, interventions need to fit with school need and ethos as well as be viewed as leading to positive outcomes for CYP [13]. Such ‘Motivation and congruence’ at the level of the school organisation is facilitative to implementation. However, systems may have difficulty prioritising early intervention when there is limited service provision for CYP presenting with more complex needs.

### Limitations

Limitations were present in the analysis of qualitative data; although data sufficiency was obtained, there is potential for the on-going emergence of themes because of the progressive nature of implementation. Full and sustainable implementation is considered to take two to four years, yet the current study was completed within an eight-month period over the initial implementation. In addition, the current study did not include interviews with the wider school system, parents or CYP who are key stakeholders in the implementation.

While the lead researcher’s role and reflexivity in the implementation was considered, it is likely that their background influenced the way in which data was interpreted. Feedback of themes did not occur due to the constraints of the school holidays and potential reporting or selection bias may have also been introduced by voluntary participation. For example, participants’ responses and, therefore, themes, may have been biased by social desirability. Moreover, during sampling, those practitioners who were not able to implement LIAM were reluctant to be involved, meaning that some potential barriers to implementation were not captured. While LIAM is a national project, the scope of the findings is taken from a single health board and findings may not all be applicable to those involved in the wider project and where the context of implementation differs. In addition, although coaching was in place to promote fidelity to the model, there was no formal measure of fidelity to examine this dimension of implementation.

#### Implications for Future Research and Clinical Practice

These findings suggest that LIAM may be generalised beyond the context of its application, although further replication is needed, along with exploration of later stages of LIAM implementation and its impact on the wider system (e. g. schools and mental health services).

Future research designs may include relational quantitative analyses to establish whether implementation variability in LIAM is predictive of outcome variability and to identify critical intervention components, taking into account the practitioner characteristics (e.g. self-efficacy and previous experience) and CYP baseline severity or diagnosis (e.g. ASD). More research is also needed on the aspects of implementation (e. g. fidelity, dose, and acceptability) and how these interact.

The manualised nature of LIAM, it’s acceptably across stakeholders, as well as the training and coaching model, has utility in promoting skill development for practitioners as well as reducing symptoms of anxiety in CYP line with the aims of the intervention. Coaching emerged as particularly important for on-going skill development, encouragement and sustaining implementation when faced with barriers. Factors relating to staff selection are also implicated to promote practitioner self-efficacy.

A key part of the implementation process is the intervention passing through critical feedback loops [7, 13, 16] and data-driven refinement over time. Future implementation efforts would benefit from collaborating with the whole school system, promoting psychological awareness and knowledge of anxiety in CYP and intervention dissemination and targeting based on the current school needs (e. g. reviewing the exclusion criteria around self-harm and ASD).

### Summary

The impact of implementation variability on outcomes is established, yet the literature on understanding implementation processes for cognitive behavioural school-based interventions for mental health and well-being is sparse. The current study supports the findings that school-based implementation is a complex, dynamic process involving multiple stakeholders and numerous interactive factors which act as facilitators and barriers. However, there is a need for service planning to consider and integrate all of these aspects in order to move towards sustained and responsive implementation.
